# Supplementary material for: Snowball: a novel gene family required for developmental patterning of fruiting bodies of mushroom-forming fungi (Agaricomycetes)
Source: mSystems. 2024 Feb 9;9(3):e01208-23. doi: 10.1128/msystems.01208-23 (PMC10949477; doi:10.1128/msystems.01208-23)
Supplement: Supplemental Figures — Figures S1 to S7 and captions for the supplemental tables. [file msystems.01208-23-s0001.pdf]

Supporting information for

Snowball: a novel gene family required for developmental patterning in fruiting bodies of mushroom-forming fungi (Agaricomycetes)

Csenge Földi[1,2], Zsolt Merenyi[1], Bálint Balázs[1], Árpád Csernetics[1], Nikolett Miklovics[1], Hongli Wu[1], Botond Hegedüs[1], Máté Virágh[1], Zhihao Hou[1], Xiao-Bin Liu[1], László Galgóczy[1,3], László G. Nagy[1]*

[1] Synthetic and Systems Biology Unit, Institute of Biochemistry, HUN-REN Biological Research Centre,, Temesvári krt. 62, Szeged H-6726, Hungary
[2] Doctoral School of Biology, Faculty of Science and Informatics, University of Szeged, Szeged H-6726, Hungary
[3] Department of Biotechnology, Faculty of Science and Informatics, University of Szeged, Szeged H-6726, Hungary

* Corresponding author (lnagy@fungenomelab.com)

Contents:

- Supplementary figures 1-7.
- Legends for supplementary tables

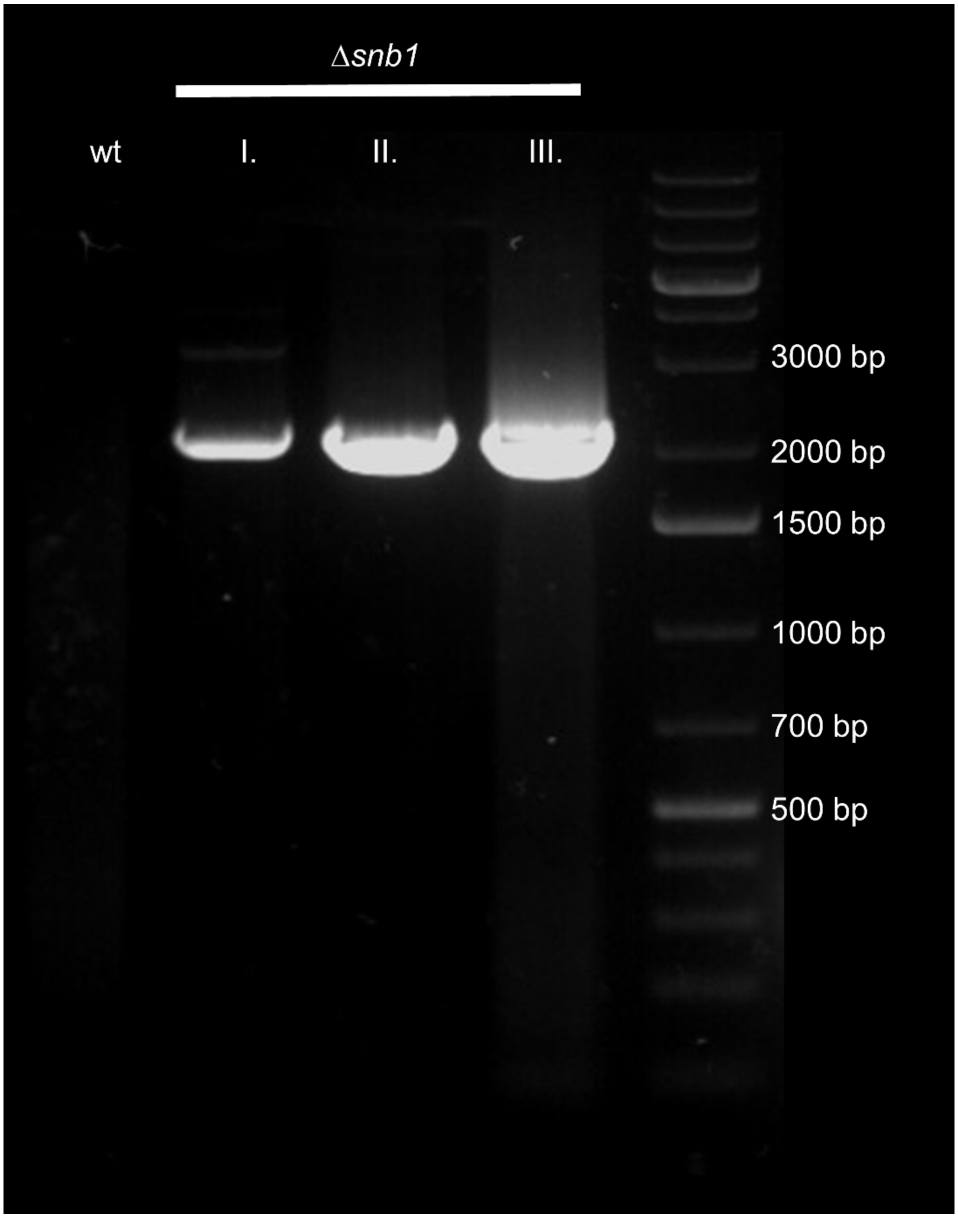

***Fig. S1**. - PCR analysis of* snb1 *knockout strains*

For the primer pair snb1_out_check_fwd/pab1_inner_250bp_rev, the expected amplicon size if the repair template integrated into the Cas9 cleavage site is 2090bp. I., II. and III. are corresponding to the three obtained Δ*snb1* strains.

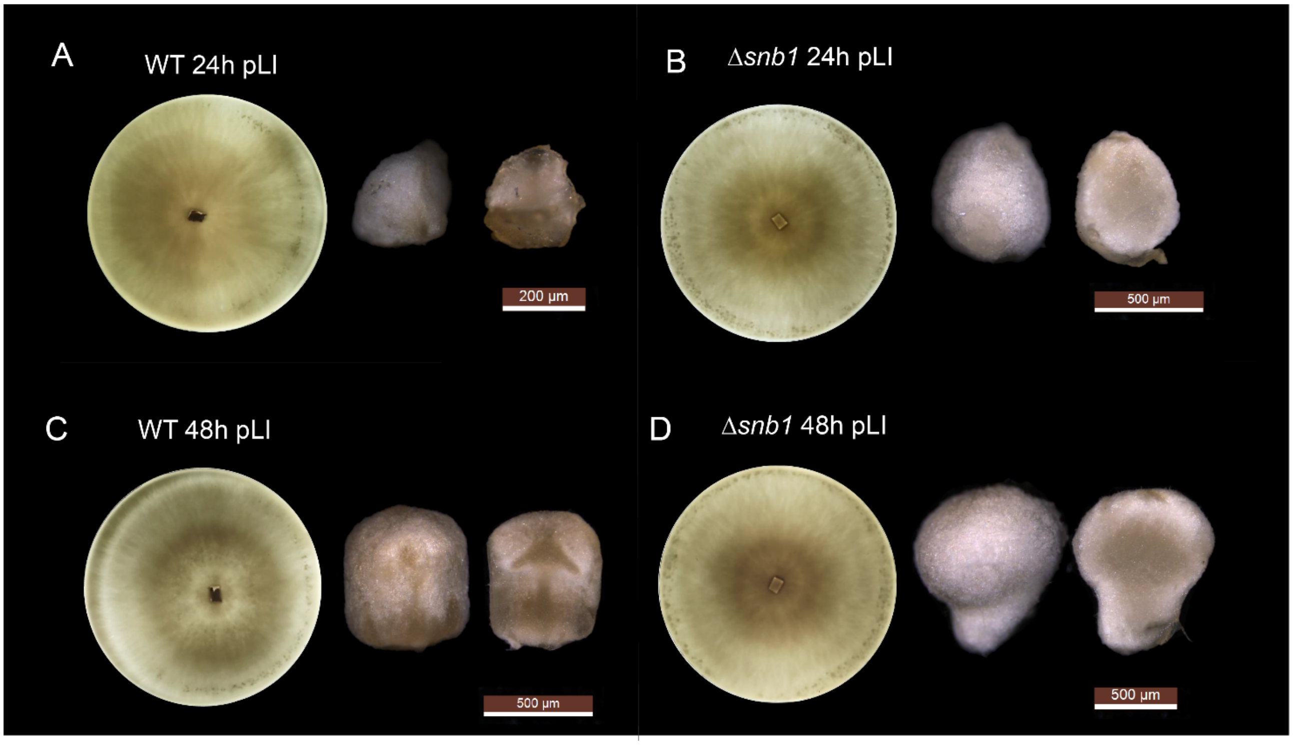

Fig. S2. *- Time points sampled for RNA-Seq experiments showing the morphology of wt and Δsnb1 at early developmental stages*. Hyphal knots **(A, B)** and stage 1 primordia **(C, D)** after 24 hours and 48 hours of light induction, respectively. Scale bars are shown under the corresponding stages.

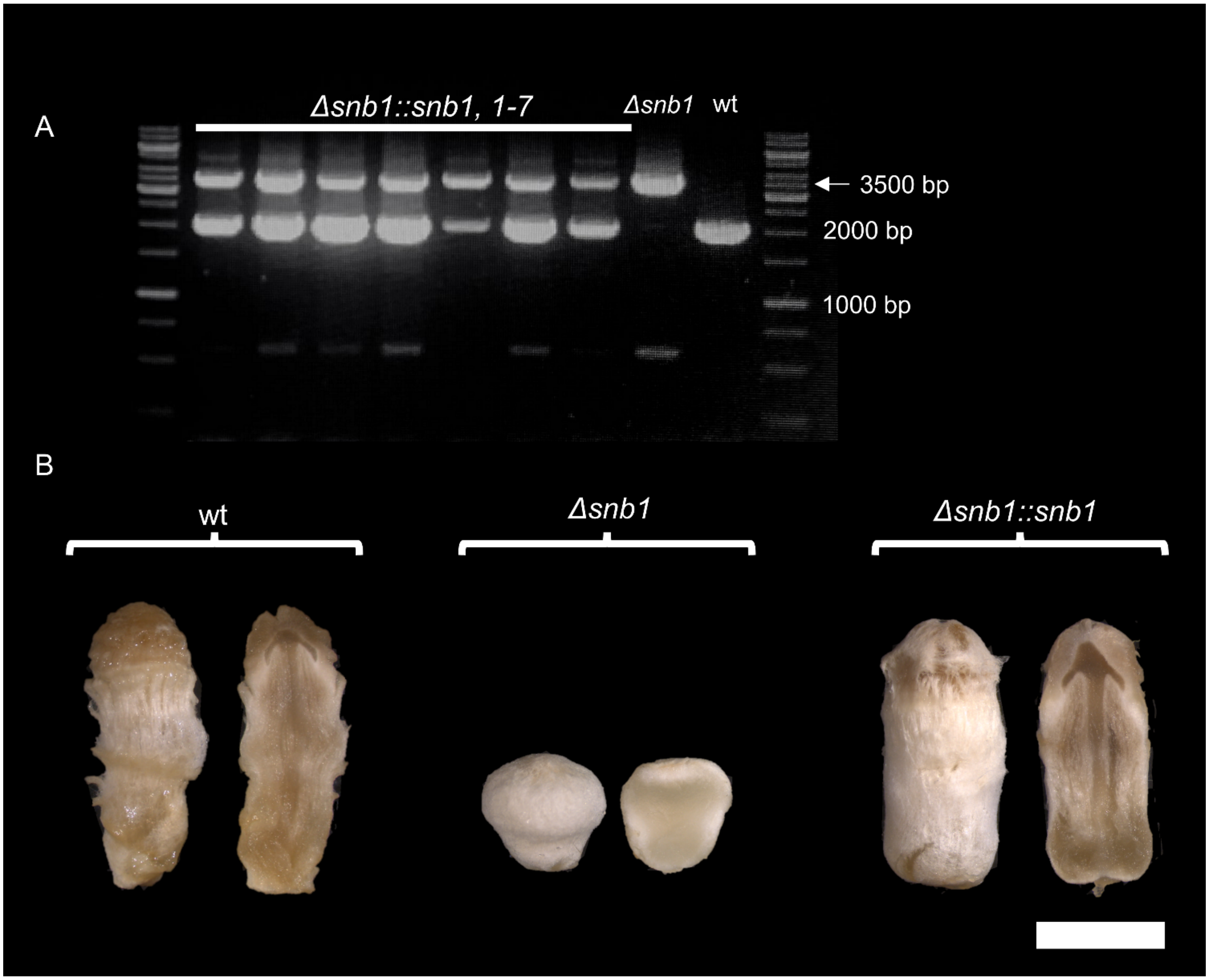

Fig. S3. *- Complementation of Δ*snb1 *strain by the wild type gene.* **A)** PCR validation of gene complementation using an inner primer pair of *snb1* (snb1_inner_fwd/snb1_inner_rev). The expected size of Δ*snb1* is larger due to the insertion of *pab1* gene (expected amplicon size in the presence of the intact *snb1* gene is 2122bp, expected amplicon size in the presence of disrupted *snb1* gene is 3602bp). **B)** Cross sections of developing fruiting bodies of wt, Δ*snb1,* and complemented *Δsnb1 C. cinerea* strains three days post-light induction (pLI). Strains were grown on YMG medium with halved glucose content at 28°C. Scale bar = 2mm.

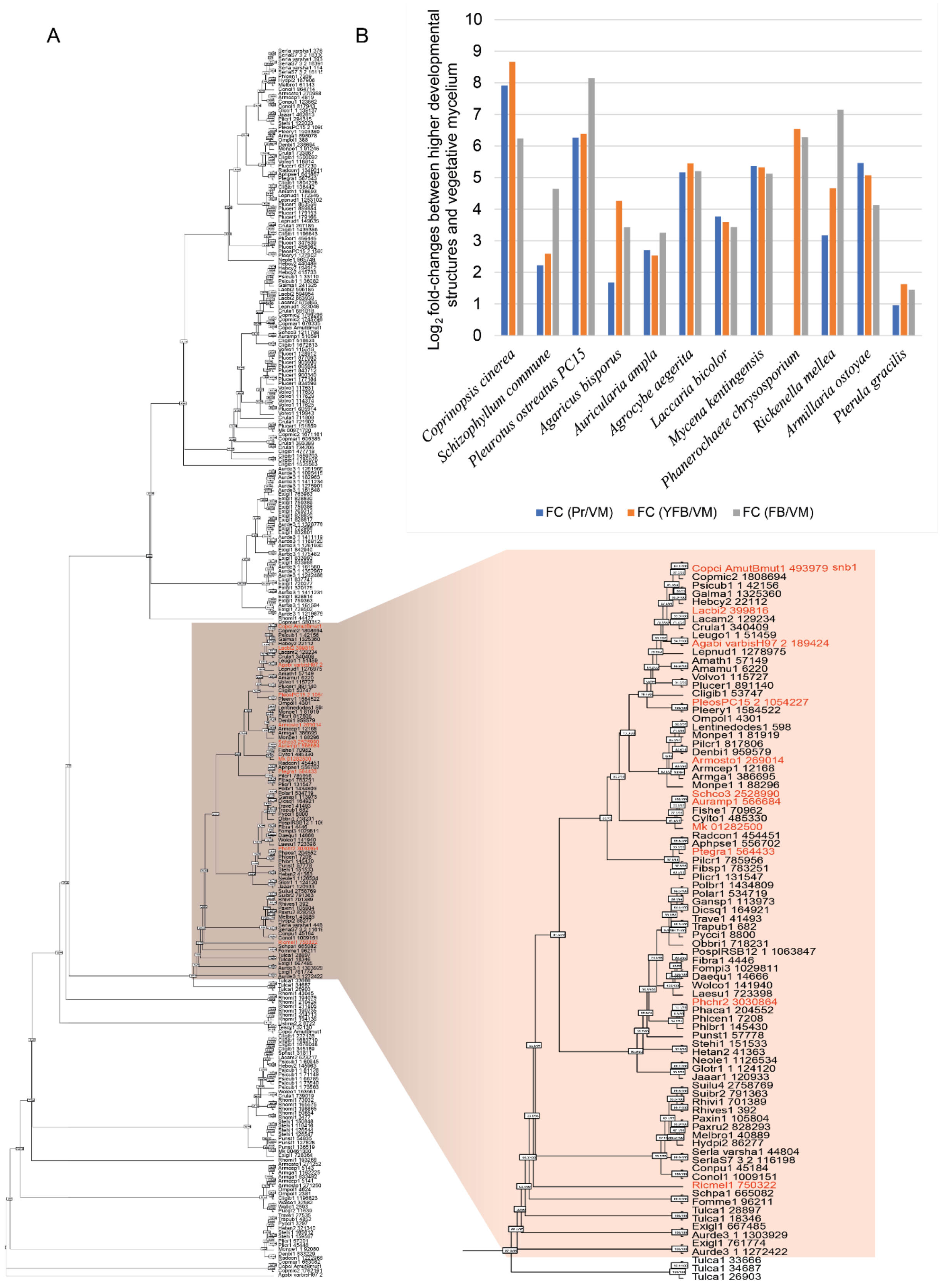

Fig. S4. - **A)** *Phylogenetic tree of SNB1 family.* Orthogroup with orthologs of SNB1 are highlighted with brownish background. Orthologs with red text are represented in Fig. S4/b. **B)** Log_2 fold-change differences in expression of *C. cinerea* SNB1 and its orthologues in further agaricomycetes species between vegetative

mycelia (VM) and different developmental stages of fruiting body (Pr: primordium, YFB: young fruiting body, FB: matured fruiting body).

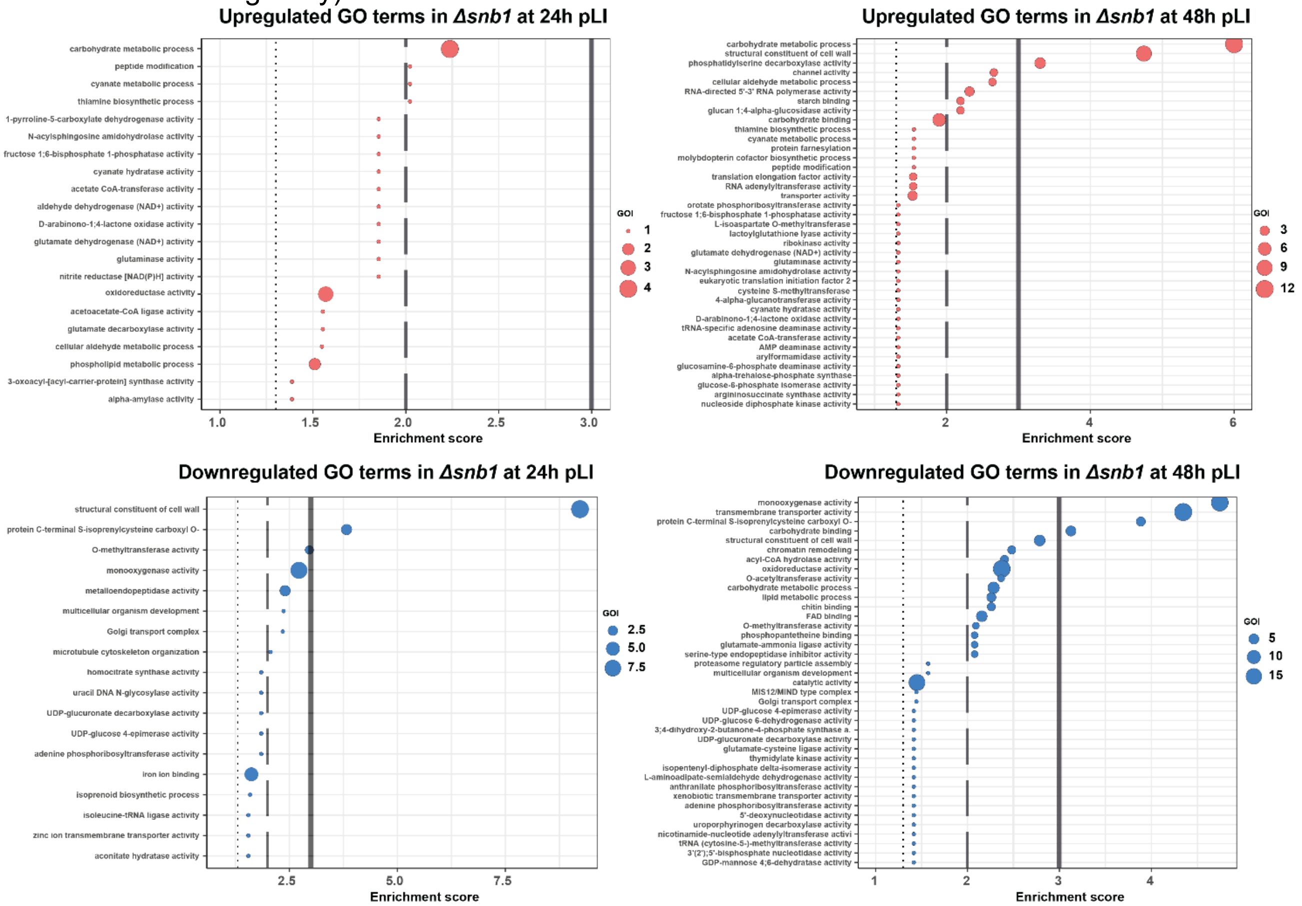

Fig. S5. - Enriched Gene Ontology (GO) terms in Δ*snb1* compared to wt 24h and 48h pLI. Cutoff lines are drawn at enrichment scores corresponding to *p*=0.05, p=0.01, and *p*=0.001 (from left to right). GO terms are ordered by Kolmogorov–Smirnov p-values. See Table S3. for details on GO enrichment.

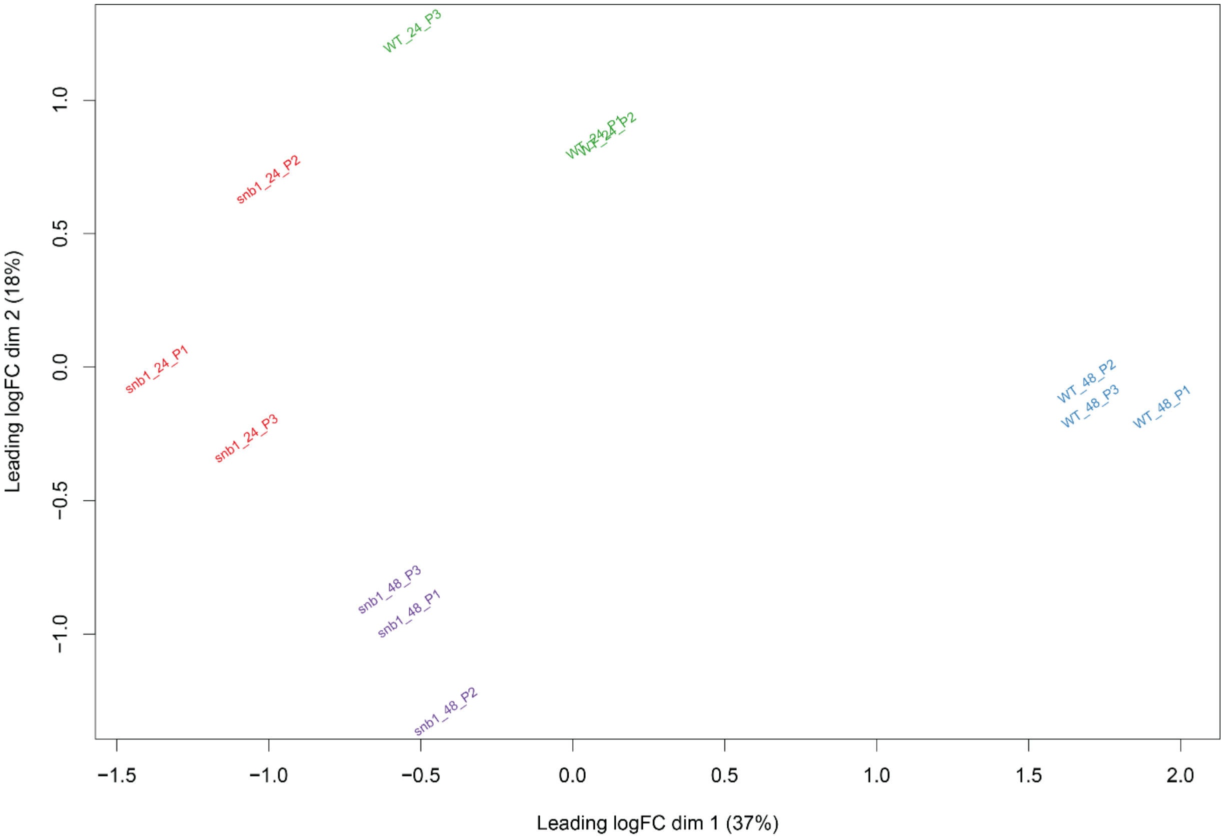

Fig. S6. *- MDS-plot of the RNA-sequencing results.*

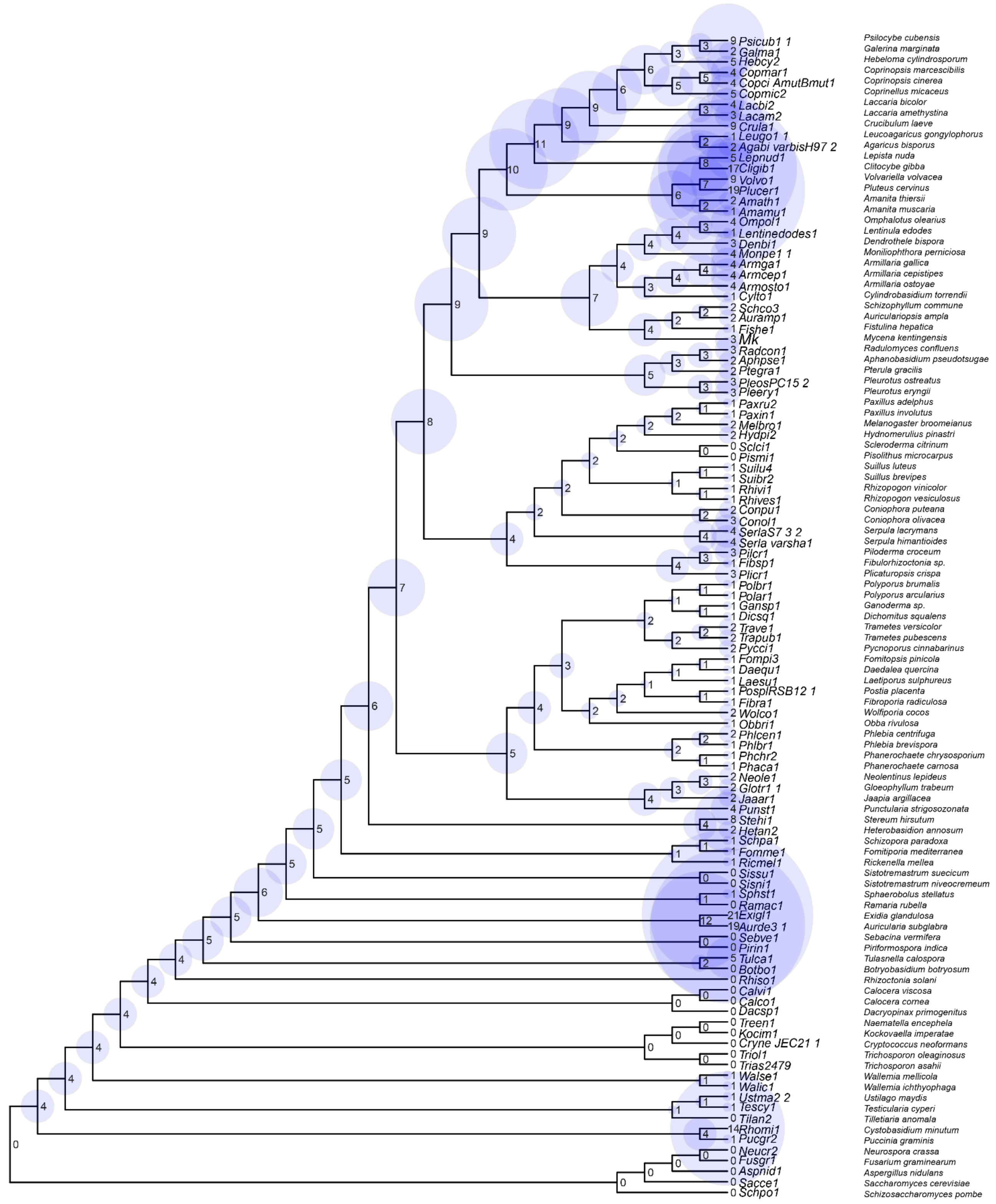

Fig. S7. *- Inferred ancestral copy numbers of the SNB1 homologs in the proteome of 109 examined species.* The size of the circles is proportional to the number of copy numbers.

Table contents:

Table S1. - List of primers used in this study.

Table S2. - All differentially expressed genes (DEGs) from the comparison of wild-type and *Δsnb1* mutant strains after 24h and 48h post-light induction (pLI).

Table S3. - Detailed list of enriched Gene Ontology (GO) terms in *Δsnb1* compared to wt 24h and 48h pLI.

Table S4. - Mapping statistics for *Δsnb1* and wild type.
